# Impact of COVID-19 pandemic on the Jordanian eating and nutritional habits

**DOI:** 10.1016/j.heliyon.2022.e09585

**Published:** 2022-05-30

**Authors:** Almu'atasim Khamees, Sajeda Awadi, Shireen Rawashdeh, Muna Talafha, Jamal Bani-Issa, Mohammad Ali S. Alkadiri, Mazhar Salim Al Zoubi, Emad Hussein, Fadi Abdel Fattah, Ibrahim H. Bashayreh, Mohannad Al-Saghir

**Affiliations:** a Faculty of Medicine, Yarmouk University, P.O Box 566, 21163, Irbid, Jordan; b Department of Statistics, Faculty of Sciences, Yarmouk University, Jordan; c Department of Food Science and Human Nutrition, A'Sharqiyah University, Ibra, Oman; d Management Department, A'Sharqiyah University, Ibra, Oman; e Nursing Department, Fatima College of Health Sciences, Al-Ain Campus, Abu-Dhabi, United Arab Emirates; f Department of Biological Sciences, Ohio University, Zanesville, OH, 43701, USA

**Keywords:** COVID-19, SARs-Cov-19, Health, Life style, Nutrition

## Abstract

Since the emergence of the COVID-19 pandemic, variable measures have been implemented to control the invasion of SARS-CoV-19 worldwide. Some of these measures included lockdowns for several months in some countries. In Jordan, various protocols have been implemented to deal with the epidemic, such as border closures, closures and local protocols for open days until we reach the partial opening in mid-2021. These measures and protocols have affected all sectors in the country, including the education system and the economy. In addition, lifestyle is one of the measurement issues that have been affected by government regulations during the COVID-19 pandemic. In this study, we aimed to investigate the impact of the COVID-19 pandemic on the eating and nutrition habits of the Jordanian population. An online sectional survey was built using Google Forms from Google. Responses were collected from November 12, 2020, through November 24, 2020. Researchers submitted a total of 2,511 responses. The results showed that there was a significant decrease in sugar intake (P = <0.005) with no critical fat and oil intake (P = 0.12). There was a significant change in daily consumption of fruits, vegetables, garlic, onions, and ginger, and downloads of health-related applications and supplements (P < 0.005). In addition, there was an increase in appetite, weight and number of daily meals. These changes are attributed to the extra free time due to lockdown and studying or working from home has significant impacts. However, 31.4% of respondents reported that the limited economic availability of food products and the closure of restaurants and cafes lead to a healthier lifestyle. Only 26.2% have been influenced by family members, friends, doctors, or social media to change their eating habits during the pandemic.

## Graphical abstract

**Figure:**
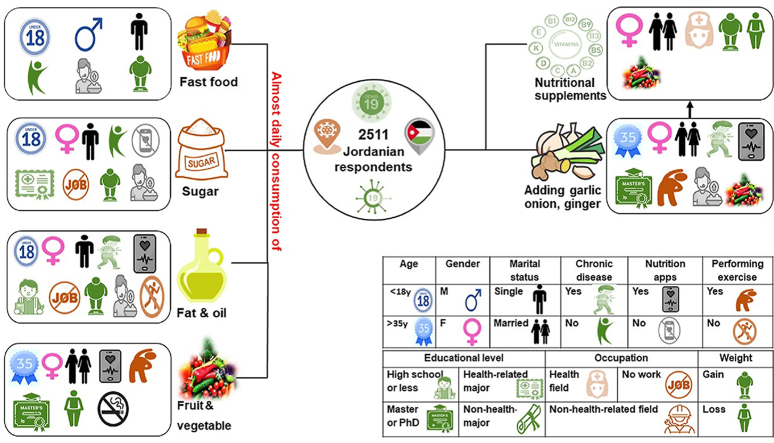

The study population included 2511 responders and the results showed the significant variables that are associated with nutritional status during the COVID-19 pandemic in Jordan (the tables represent the key).

## 1 Introduction

During the period between the appearance of the first cases of COVID-19 in late 2019 and March 11, 2020 - the date when the World Health Organization declared SARS-COVID19 a global pandemic - countries around the world have imposed lockdown laws and taken various measures to control the spread of the virus [1]. In Jordan, the first case of COVID was confirmed on March 2, 2020 [2]. Shortly after this date, the government announced a complete nationwide lockdown. Travel of all kinds was also banned, while all sectors and institutions were obligated to perform their duties online except for health sectors, stores and a few government administrative offices. Social isolation was the norm for nearly forty-five days [3, 4, 5]. During this period, people's daily habits changed suddenly, and a change in their lifestyles was inevitable.

Full or partial lockdown measures resulted in more people adopting inactive behaviors [6, 7, 8, 9]. Many people developed poor eating habits due to limited access to nutritious, fresh foods or stress-related eating behaviors (e.g., binge eating).8 Furthermore, there was a significant increase in carbohydrate consumption, intake of sweets, fast food, tea consumption, increased number of snacks during the day, and a decrease in the intake of seafood [10, 11, 12]. On the other hand, there was an increase in some healthy diets such as increased fruit, vegetables, protein intake, and a decrease in consumption of alcohol [10].

Moreover, many people have decided to strengthen their immune system to fight infection, by making healthier diet choices and taking dietary supplements [13, 14, 15, 16]. Maintaining a healthy weight has also become a goal for some people as obesity has been proved to be a nasty prognostic factor in the course of a COVID-19 infection [17]. Although WHO experts have issued recommendations regarding nutrition and physical activity for people to follow to preserve their health during this challenging time [18, 19, 20], the economic and psychosocial impacts of this pandemic are complex, and people continue to struggle to adjust to all the new norms.

This study aims to examine whether and how the people of Jordan have changed some of their health-related behaviors during the COVID-19 pandemic. The measures we seek to monitor include diets, dietary supplement use, and weight changes.

## 2 Materials and methods

In the wake of the COVID19 pandemic, people around the world have witnessed a major change in how they go about their daily lives. An essential aspect of human well-being is diet. In light of this, we conducted an online survey to study the eating behaviors of the Jordanian population during the national lockdown that Jordan underwent in the first months of 2020. The aim was to discover whether and how people's eating habits changed under the newly imposed restrictions. The study wanted to reveal patterns of pro-health versus unhealthy food choices and see if the pandemic is making people eat more.

### 2.1 Procedure and data collection

The current study is an online cross-sectional survey built using Google's Google Forms service. It consists of three main question sets containing all the multiple-choice questions. At the beginning of the survey, we mentioned that the data collected will only be used for this study. No personal information was obtained through any questions in the survey, and participants voluntarily filled out the survey and had the option to leave the link at any time.

The survey was primarily distributed via social media platforms, including WhatsApp, Facebook, Instagram, and via the Yarmouk University email portal to all students in the university. The researchers relied on their family members and friends to pass on the survey link to get as many respondents as possible.

The target population was Jordanians that have been residing in Jordan since the start of the pandemic. The survey is a self-administered questionnaire. It was written in the Arabic language, the mother tongue of Jordanians. Responses were collected starting from November 12, 2020, until November 24, 2020. The researchers yielded a total of 2511 responses.

Inclusion and exclusion criteria: All participants who live in Jordan since the start of the pandemic can complete the questionnaire in the Arabic language and give online consent participants were included in this study.

### 2.2 Measures

#### 2.2.1 Socio-demographics

Participants were asked to specify their age, gender, educational level, and occupation. The story of education was sub-classified into "High school diploma or still in school," "Diploma or bachelor's degree in a health-related major," "Diploma or bachelor's degree in non-health-related major," and "Master's degree or Ph.D." As for occupation, we divided participants into those working in a health-related field, those working in a non-health-related field, and those who did not work.

#### 2.2.2 Food choices and eating-related behaviors

The consumption patterns of four food groups were studied. These were junk food, sugar, fats, oils, fruits and vegetables. Participants were asked to indicate the frequency of their intake of these nutrients before and after the pandemic began to see if and how it had changed during the newly emerging conditions. They had to choose four frequency classes; in sequential order were: "almost daily", "three to four times a week", "once a week", "once a month or less".

The number of daily consumed meals, the number of meals eaten during nighttime, and how the pandemic affected these numbers were assessed. Questions regarding changes observed by participants in their weight and appetite were posed. Participants were also asked about their use of social media platforms to access dietary and nutrition-related content both before and after the start of the pandemic.

We wanted to determine the attitude of the study sample toward taking dietary supplements and whether the general population has changed their views about taking them. We also asked participants about adding well-known natural remedies such as onions, garlic and ginger to their diets.

## 3 Results

### 3.1 Socio-demographic characteristics

**Figure 1:**
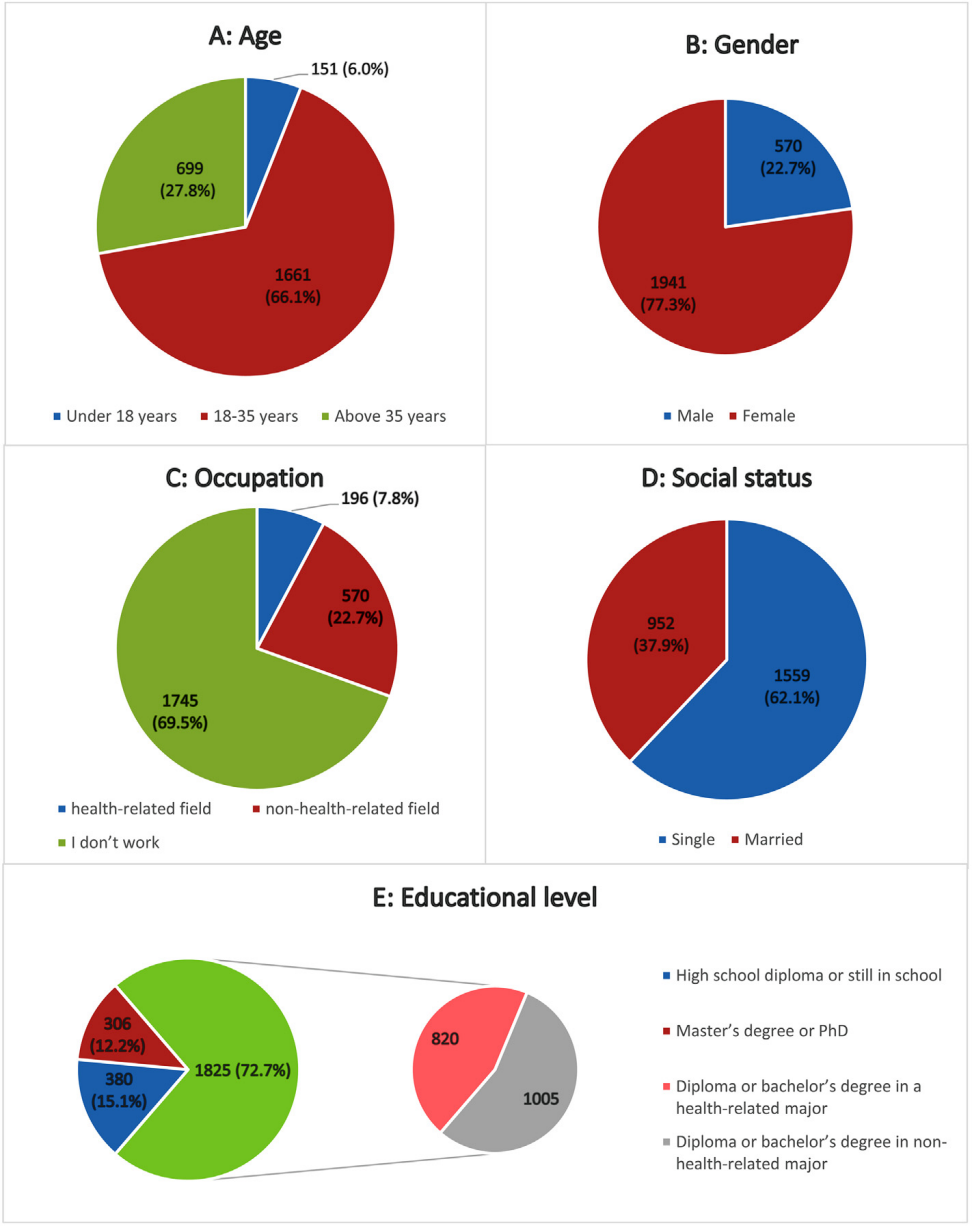
Demographic data of the Jordanian respondents who participate in this study (n = 2511). **A:** Representing age of respondent (distributed as less than 18 years, between 18 – 35 years, and above 35 years). **B:** Representing gender groups (male and female). **C:** Representing occupations of the cohort (health-related, non-health-related, and not working. **D:** Representing social status (single or married). **E:** Representing educational level (High school diploma or still in school, Diploma or bachelor's degree in a health-related major (Nutrition, Medicine, Dentistry, Allied health sciences), Diploma or bachelor's degree in non-health-related major, Master's degree or PhD). The majority of respondents were (66.1%) in the 18–35 years category, 77.3% were females, 69.5% were with no occupation, 62.1% were single marital status, and 72.7% had a diploma or bachelor's degree.

A total of 2511 respondents from all governorates in Jordan completed the questionnaire. Table 1 shows the demographic characteristics of the respondents. Out of them, 1941 were females (77.3%), 1661 (66.1%) were in the age range between 18 and 35 years, 1559 (62.1%) were single, and 1873 (74.6%) lived in a city. The majority of respondents had a diploma or bachelor's degree in 1825 (72.7%), and 1005 of them (55.1%) had a diploma or bachelor's degree in a non-health-related major. Five hundred seventy respondents (22.7%) worked in a non-healthy related field and 196 (7.8%) in a health-related area (Figure 1).

**Table 1:** Demographic characteristics for the participants (n = 2511).

**Age**	
Under 18 years	151 (6.0%)
18–35 years	1661 (66.1%)
Above 35 years	699 (27.8%)
**Gender**	
Male	570 (22.7%)
Female	1941 (77.3%)
**Social status**	
Single	1559 (62.1%)
Married	952 (37.9%)
**Number of household members**	
3 or less	416 (16.6%)
4–6	1316 (52.4%)
7 or more	779 (31.0%)
**Educational level**	
High school diploma or still in school	380 (15.1%)
Diploma or bachelor's degree in a health-related major	820 (32.7%)
Diploma or bachelor's degree in non-health-related major	1005 (40.0%)
Master's degree or PhD	306 (12.2%)
**Occupation**	
health-related field	196 (7.8%)
non-health-related field	570 (22.7%)
I don't work	1745 (69.5%)
**Place of residence**	
City	1873 (74.6%)
Village	615 (24.5%)
Refugee camp	23 (0.9%)
**Do you suffer from any chronic illnesses?**	
Yes	280 (11.2%)
No	2231 (88.8%)
**Do you currently perform any form of physical exercise?**	
Yes	1103 (43.9%)
No	1408 (56.1%)
**Do you currently smoke (this includes regular cigarettes, electronic cigarettes, hookah, etc.)?**	
Yes	478 (19.0%)
No	2033 (81.0%)

#### 3.1.1 Food consumption behavior

The survey was designed to explore the consumption patterns of four food groups: fast food, sugar, fat and oils, and fruits and vegetables. Respondents were asked to indicate the frequency of their intake of these foodstuffs both before and after the start of the pandemic to see if and how it changed throughout the newly arising circumstances. They had to choose four frequency categories; these in consecutive order were: "Almost daily," "Three to four times a week," "Once a week," and "Once a month or less."

**Figure 2:**
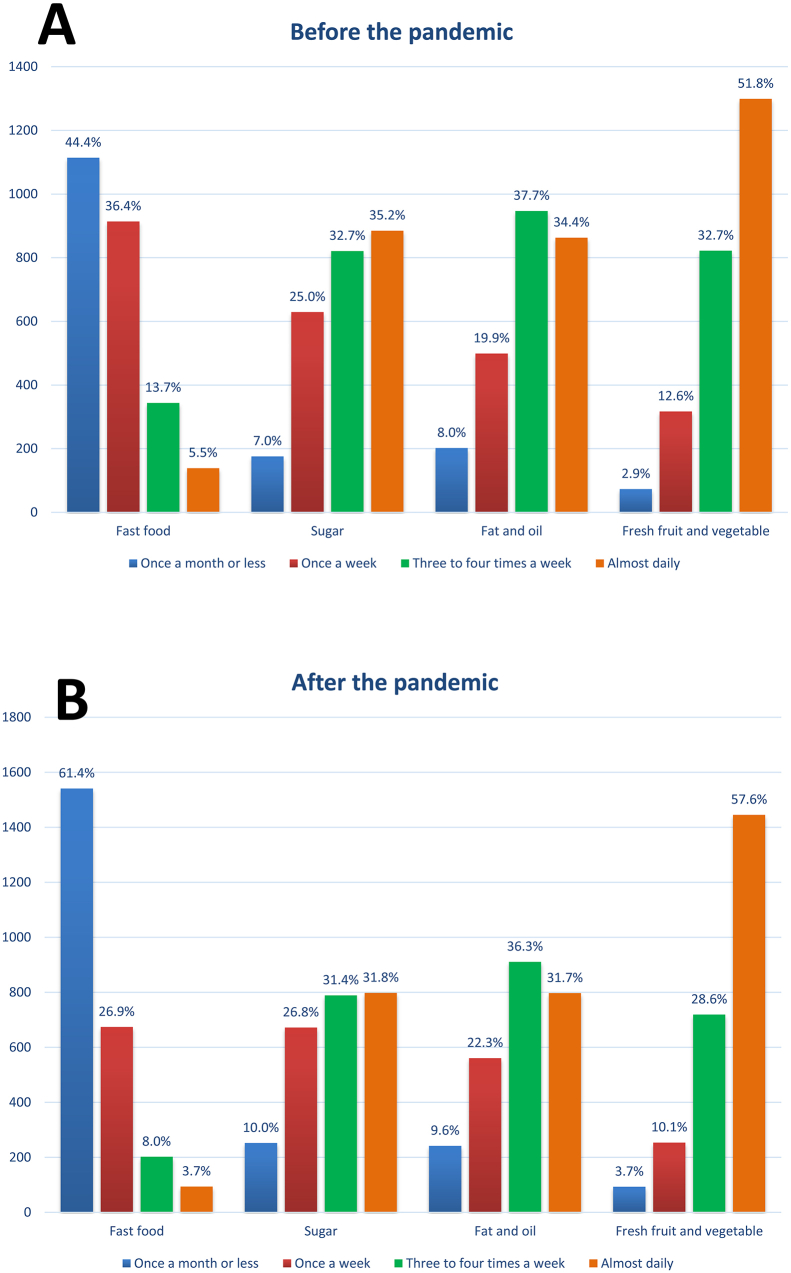
Food consumption behavior before (A) and after (B) the start of the pandemic. The respondents were asked to indicate the frequency (almost daily, three to four times a week, once a week, and once a month or less) of their intake of four food groups (fast food, sugar, fat and oils, fruits and vegetables) before and after the start of the pandemic.

For instance, when the respondents were asked about the frequency of their fast-food intake, 483 (19.2%) stated that they consumed fast food three to four times or almost daily before the pandemic, and this consumption significantly decreased by 1.6-fold after the pandemic to be 295 (11.7%) (P-value < 0.05). However, there is a significant reduction in the sugar intake (P-value = 0.000) on an almost daily basis from 885 (35.2%) before the pandemic to 798 (31.8%) following it. Also, the number of respondents who stated that they consumed sugar once monthly rose from 176 (7.0%) before the pandemic to 252 (10.0%) after the pandemic (P-value = 0.000). Correspondingly for fat and oil intake, there is a statistical association in reduction of daily consumption of fat and oil (P-value = 0.000), which decreased from 863 (34.4%) before the pandemic to 797 (31.7%) after the start of the pandemic. On the other hand, the number of respondents who consumed fat and oil once monthly slightly rose from 202 (8.0%) before the pandemic to 242 (9.6%) after the start of the pandemic (P-value = 0.000). There is a significant change in daily intake of fruits and vegetables (P-value = 0.000) which was 1299 (51.8%) before and 1445 (57.6%) after the pandemic (Figure 2).

Notwithstanding, the change in fast food consumption, sugar, fat, oil, fresh fruit, and vegetable before and after the pandemic was significantly associated with demographic factors like age, gender, social status, educational level, and occupation (P-value for all <0.05) [Tables 2, 3, 4, 5, and 6].

Respondents who consumed almost daily fast food since the start of the pandemic were significantly associated with young age less than 18 years, male gender, single social status, increased number of daily meals, nighttime meals, increased appetite (P-value for all = 0.000), gain weight (P-value = 0.001), free of chronic disease (P-value = 0.003), and having 3 or fewer household members (P-value = 0.005). However, there was no association with educational level (P-value = 0.117), occupation (P-value = 0.186), performing physical activity (P-value = 0.139), and downloading any applications or starting to follow any social media accounts concerning healthy lifestyle and nutrition (P-value = 0.770) [Tables 2, 3, 4, 5, and 6].

Almost daily consumption of fast food was 2.4-fold more in males, 2-fold more in singles, and 2.2-fold more among smokers compared with females, married, and non-smokers respondents, respectively (P-value for all was 0.000) [Tables 2 and 3]. Moreover, 798 (31.8%) respondents had almost daily sugar intake since the start of the pandemic. This behavior was significantly associated with young age less than 18 years (P-value = 0.000), female gender (P-value = 0.039), single social status (P-value = 0.000), having 7 or more household members (P-value = 0.031), having Diploma or bachelor's degree in a health-related major (P-value = 0.001), having no occupation (P-value = 0.000), free of chronic disease (P-value = 0.000), increased number of day meals (P-value = 0.000), nighttime meals (P-value = 0.000), increased in appetite (P-value for all = 0.000), gain weight (P-value = 0.000), not performing physical activity (P-value = 0.000), and not downloading any applications or following any social media accounts concerning healthy lifestyle and nutrition (P-value = 0.000). On the other hand, smoking status was not associated with daily sugar intake (P-value = 0.218) [Tables 2, 3, 4, 5, and 6].

Regarding fat and oil intake, 797 (31.7%) respondents had almost daily intake since the start of the pandemic. Respondents who were less than 18 years (P-value = 0.000), female gender (P-value = 0.001), single social status (P-value = 0.003), having 7 or more household members (P-value = 0.028), having high school diploma or still in school (P-value = 0.000), having no occupation (P-value = 0.003), having a chronic disease (P-value = 0.000), increased number of day meals (P-value = 0.000), nighttime meals (P-value = 0.000), increased in appetite (P-value for all = 0.000), gain weight (P-value = 0.000), not performing physical activity (P-value = 0.000), and not downloading any applications or following any social media accounts concerning healthy lifestyle and nutrition (P-value = 0.008) were significantly associated with daily fat and oil intake. Otherwise, there was no associated with smoking status (P-value = 0.855) [Tables 2, 3, 4, 5, and 6].

More than half of the respondents (n = 1445) (57.6%) consumed almost daily fresh fruit and vegetable since the start of the pandemic. This healthy behavior was significantly associated with being more than 35 years old, female gender, married social status, having a master's degree or Ph.D., performing physical exercise, losing weight, or no change in their weight, no change in the number of daily meals, or night meals, downloading any applications or starting to follow any social media accounts concerning healthy lifestyle and nutrition (P-value for all = 0.000), and non-smokers (P-value = 0.003). However, there was no significant association with the number of household members, occupation, having a chronic illness, and changes in appetite (P-value was 0.253, 0.190, 0.403, and 0.051, respectively) [Tables 2, 3, 4, 5, 6, and 7].

#### 3.1.2 Weight and appetite during the pandemic

To understand other eating habits of individuals in the study sample, a couple of questions were posed regarding the number of meals respondents consumed in a day and whether the number of meals they had during nighttime had increased during the pandemic. There was 945 (37.6%) reported an increase in their appetite, and 618 (65.4%) of them had increased weight, 844 (33.6%) respondents noted an increase in the number of meals eaten in a day, and 549 of them (65.0%) gained weight, 1005 (40.0%) responded that they have noticed an increase in the number of meals they consumed during the night and 583 of them (58.0%) had increased weight. However, 913 (36.4%) of all respondents noticed some weight gain (Tables 2, 3, 4, 5, and 6).

Respondents who gained weight since the starting of the pandemic were significantly associated with age less than 18 years (P-value = 0.000), male gender (P-value = 0.003), married social status (P-value = 0.000), having 7 or more household members (P-value = 0.026), having diploma or bachelor's degree in non-health-related major (P-value = 0.011), working in a non-health-related field (P-value = 0.000), not performing physical exercise (P-value = 0.000), increased number of day meals (P-value = 0.000), and nighttime meals (P-value = 0.000). On the other hand, there was no significant association with having chronic disease (P-value = 0.345), smoking status (P-value = 0.282), and downloading any applications or following any social media accounts concerning healthy lifestyle and nutrition (P-value = 0.052) [Tables 2, 3, 4, 5, 6, 9, and 10].

Also, increasing in appetite since the starting of the pandemic were significantly associated with respondents who were less than 18 years (P-value = 0.000), female gender (P-value = 0.001), single social status (P-value = 0.000), having diploma or bachelor's degree in health-related major (P-value = 0.027), having no occupation (P-value = 0.012), free chronic disease (P-value = 0.047), not performing physical exercise (P-value = 0.020), gained weight (P-value = 0.000), increased number of day meals (P-value = 0.000), and nighttime meals (P-value = 0.000). However, there was no significant association with number of household members (P-value = 0.193), smoking status (P-value = 0.236), and downloading any applications or following any social media accounts concerning healthy lifestyle and nutrition (P-value = 0.277) [Tables 2, 3, 4, 5, 6, 9, and 10].

#### 3.1.3 Alternative medicine behaviors during the pandemic

Since early in the pandemic, a large body of new literature has surfaced on the use of complementary and alternative medicine for boosting immune function to prevent or mitigate a SARs-Cov19 infection. Among the circulating alternative medicine recommendations on social media outlets, especially in Jordan, was adding ingredients like garlic, onion, and ginger to one's daily diet. Our questionnaire asked respondents whether they included at least one of the ingredients mentioned above in their diets, like garlic, onion, and ginger. We found that 65.5% (1645) of the total respondents gave a positive response. We found that there was a significant solid association (P-value of 0.000) between those who have increased the intake of the ingredients mentioned above and those who have had a high intake of fresh fruits and vegetables since the beginning of the pandemic as 1000 of the 2511 respondents were consuming fresh fruits and vegetables almost daily. Likewise, there was a strong association between respondents who consumed fast food once monthly or less and sugar intake once weekly (P-value = 0.000), while no association with the frequency of fat and oil intake (P-value = 0.594) [Table 8].

Also, respondents above 35 years, female gender, married social status, master's degree, or Ph.D. (P-value for all 0.000), having a chronic disease (P-value = 0.013), performing physical exercise (P-value = 0.020), increased appetite (P-value = 0.009), and downloading any applications or following any social media accounts concerning healthy lifestyle and nutrition (P-value = 0.000) were strongly associated with increased intake of ingredients like garlic, onion, and ginger. On the other hand, adding these ingredients was not associated with occupation, weight changes, the number of meals during the daytime, or nighttime, and smoking status (P-value was 0.075, 0.187, 0.141, 0.155, and 0.604, respectively) [Tables 2, 3, 4, 5, 6, 7, 8, 9, and 10].

**Table 2:** Food choices and eating-related behaviors before and during COVID-19 lockdown according to the age group (n = 2511).

		Age				P value	Chi square test (P-value)
		Under 18 years	18–35 years	Above 35 years	Total		
Fast food intake (before the start of the pandemic)	Once a month or less	75 (3.0%)	592 (23.6%)	447 (17.8%)	1114 (44.4%)	**0.000**	**0.003**
	Once a week	51 (2.0%)	661 (26.3%)	202 (8.0%)	914 (36.4%)		
	Three to four times a week	21 (0.8%)	290 (11.5%)	33 (1.3%)	344 (13.7%)		
	Almost daily	4 (0.2%)	118 (4.7%)	17 (0.7%)	139 (5.5%)		
Fast food intake (after the start of the pandemic)	Once a month or less	89 (3.5%)	937 (37.3%)	515 (20.5%)	1541 (61.4%)	**0.000**	
	Once a week	39 (1.6%)	493 (19.6%)	142 (5.7%)	674 (26.9%)		
	Three to four times a week	15 (0.6%)	159 (6.3%)	28 (1.1%)	202 (8.0%)		
	Almost daily	8 (0.3%)	71 (2.8%)	14 (0.6%)	93 (3.7%)		
Sugar intake (before the start of the pandemic)	Once a month or less	6 (0.2%)	83 (3.3%)	87 (3.5%)	176 (7.0%)	**0.000**	**0.000**
	Once a week	34 (1.4%)	361 (14.4%)	234 (9.3%)	629 (25.0%)		
	Three to four times a week	54 (2.2%)	565 (22.5%)	202 (8.0%)	821 (32.7%)		
	Almost daily	57 (2.3%)	652 (26.0%)	176 (7.0%)	885 (35.2%)		
Sugar intake (after the start of the pandemic)	Once a month or less	11 (0.4%)	122 (4.9%)	119 (4.7%)	252 (10.0%)	**0.000**	
	Once a week	37 (1.5%)	406 (16.2%)	229 (9.1%)	672 (26.8%)		
	Three to four times a week	47 (1.9%)	539 (21.5%)	203 (8.1%)	789 (31.4%)		
	Almost daily	56 (2.2%)	594 (23.7%)	148 (5.9%)	798 (31.8%)		
Fat and oil intake (before the start of the pandemic)	Once a month or less	9 (0.4%)	112 (4.5%)	81 (3.2%)	202 (8.0%)	**0.000**	**0.000**
	Once a week	35 (1.4%)	297 (11.8%)	167 (6.7%)	499 (19.9%)		
	Three to four times a week	46 (1.8%)	664 (26.4%)	237 (9.4%)	947 (37.7%)		
	Almost daily	61 (2.4%)	588 (23.4%)	214 (8.5%)	863 (34.4%)		
Fat and oil intake (after the start of the pandemic)	Once a month or less	16 (0.6%)	129 (5.1%)	97 (3.9%)	242 (9.6%)	**0.000**	
	Once a week	31 (1.2%)	366 (14.6%)	164 (6.5%)	561 (22.3%)		
	Three to four times a week	46 (1.8%)	624 (24.9%)	241 (9.6%)	911 (36.3%)		
	Almost daily	58 (2.3%)	542 (21.6%)	197 (7.8%)	797 (31.7%)		
Fresh fruit and vegetable intake (before the start of the pandemic)	Once a month or less	10 (0.4%)	57 (2.3%)	5 (0.2%)	72 (2.9%)	**0.000**	**0.016**
	Once a week	13 (0.5%)	250 (10.0%)	54 (2.2%)	317 (12.6%)		
	Three to four times a week	45 (1.8%)	566 (22.5%)	211 (8.4%)	822 (32.7%)		
	Almost daily	83 (3.3%)	787 (31.4%)	429 (17.1%)	1299 (51.8%)		
Fresh fruit and vegetable intake (after the start of the pandemic)	Once a month or less	10 (0.4%)	64 (2.5%)	18 (0.7%)	92 (3.7%)	**0.000**	
	Once a week	8 (0.3%)	189 (7.5%)	57 (2.3%)	254 (10.1%)		
	Three to four times a week	41 (1.6%)	507 (20.2%)	171 (6.8%)	719 (28.6%)		
	Almost daily	92 (3.7%)	900 (35.9%)	453 (18.0%)	1445 (57.6%)		
(Since the start of the pandemic) have you added any of these ingredients (garlic, onion, ginger) to your diet, or increased your intake of them?	Yes	92 (3.7%)	1041 (41.5%)	512 (20.4%)	1645 (65.5%)	**0.000**	
	No	59 (2.3%)	620 (24.7%)	187 (7.4%)	866 (34.5%)		
(Since the start of the pandemic) have you noticed any change in your weight?	I have gained weight.	59 (2.3%)	604 (24.1%)	250 (10.0%)	913 (36.4%)	**0.000**	
	I have lost weight	36 (1.4%)	349 (13.9%)	88 (3.5%)	473 (18.8%)		
	My weight hasn't changed	43 (1.7%)	588 (23.4%)	330 (13.1%)	961 (38.3%)		
	I don't know	13 (0.5%)	120 (4.8%)	31 (1.2%)	164 (6.5%)		
(Since the start of the pandemic) has the number of meals you eat in a day changed?	Increased	62 (2.5%)	629 (25.0%)	153 (6.1%)	844 (33.6%)	**0.000**	
	Decreased	23 (0.9%)	301 (12.0%)	83 (3.3%)	407 (16.2%)		
	No change	66 (2.6%)	731 (29.1%)	463 (18.4%)	1260 (50.2%)		
(Since the start of the pandemic) has the number of meals you eat during nighttime increased?	Yes	68 (2.7%)	754 (30.0%)	183 (7.3%)	1005 (40.0%)	**0.000**	
	No	83 (3.3%)	907 (36.1%)	516 (20.5%)	1506 (60.0%)		
(Since the start of the pandemic) have you noticed any change in your appetite?	It has increased	67 (2.7%)	668 (26.6%)	210 (8.4%)	945 (37.6%)	**0.000**	
	It has decreased	31 (1.2%)	300 (11.9%)	81 (3.2%)	412 (16.4%)		
	It hasn't changed	53 (2.1%)	693 (27.6%)	408 (16.2%)	1154 (46.0%)		
Downloading any application or started following any social media account concerning Healthy Nutrition	Yes	37 (1.5%)	420 (16.7%)	261 (10.4%)	718 (28.6%)	**0.000**	
	No	114 (4.5%)	1241 (49.4%)	438 (17.4%)	1793 (71.4%)		
Before the pandemic, were you interested in consuming nutritional supplements like vitamins and minerals including iron and zinc?	Yes	44 (1.8%)	518 (20.6%)	229 (9.1%)	791 (31.5%)	0.612	**0.010**
	No	107 (4.3%)	1143 (45.5%)	470 (18.7%)	1720 (68.5%)		
Since the beginning of the pandemic, did you introduce nutritional supplements to your diet or increase the amount that you consume?	Yes	68 (2.7%)	740 (29.5%)	343 (13.7%)	1151 (45.8%)	0.130	
	No	83 (3.3%)	921 (36.7%)	356 (14.2%)	1360 (54.2%)		
Do you think nutritional supplements play a role in boosting the immunity to fight off diseases like COVID-19?	Yes	106 (4.2%)	1284 (51.1%)	542 (21.6%)	1932 (76.9%)	0.348	
	No	11 (0.4%)	105 (4.2%)	42 (1.7%)	158 (6.3%)		
	I do not know	34 (1.4%)	272 (10.8%)	115 (4.6%)	421 (16.8%)		
**Total**		**151 (6.0%)**	**1661 (66.1%)**	**699 (27.8%)**	**2511 (100.0%)**		

Values are expressed as number and percentage from total respondents (**n (%)**)Variables are considered significant at P-value **< 0.05** and marked in bold.

**Table 3:** Food choices and eating-related behaviors before and during COVID-19 lockdown according to the gender of participants (n = 2511).

		Gender			P- value	Chi square test (P-value)
		Male	Female	Total		
Fast food intake (before the start of the pandemic):	Once a month or less	221 (8.8%)	893 (35.6%)	1114 (44.4%)	**0.000**	**0.000**
	Once a week	181 (7.2%)	733 (29.2%)	914 (36.4%)		
	Three to four times a week	110 (4.4%)	234 (9.3%)	344 (13.7%)		
	Almost daily	58 (2.3%)	81 (3.2%)	139 (5.5%)		
Fast food intake (after the start of the pandemic)	Once a month or less	282 (11.2%)	1259 (50.2%)	1541 (61.4%)	**0.000**	
	Once a week	172 (6.9%)	502 (20.0%)	674 (26.9%)		
	Three to four times a week	78 (3.1%)	124 (4.9%)	202 (8.0%)		
	Almost daily	38 (1.5%)	55 (2.2%)	93 (3.7%)		
Sugar intake (before the start of the pandemic)	Once a month or less	57 (2.3%)	119 (4.7%)	176 (7.0%)	**0.002**	**0.000**
	Once a week	148 (5.9%)	481 (19.2%)	629 (25.0%)		
	Three to four times a week	192 (7.6%)	629 (25.0%)	821 (32.7%)		
	Almost daily	173 (6.9%)	712 (28.4%)	885 (35.2%)		
Sugar intake (after the start of the pandemic)	Once a month or less	67 (2.7%)	185 (7.4%)	252 (10.0%)	**0.039**	
	Once a week	168 (6.7%)	504 (20.1%)	672 (26.8%)		
	Three to four times a week	178 (7.1%)	611 (24.3%)	789 (31.4%)		
	Almost daily	157 (6.3%)	641 (25.5%)	798 (31.8%)		
Fat and oil intake (before the start of the pandemic)	Once a month or less	60 (2.4%)	142 (5.7%)	202 (8.0%)	**0.000**	**0.000**
	Once a week	132 (5.3%)	367 (14.6%)	499 (19.9%)		
	Three to four times a week	223 (8.9%)	724 (28.8%)	947 (37.7%)		
	Almost daily	155 (6.2%)	708 (28.2%)	863 (34.4%)		
Fat and oil intake (after the start of the pandemic)	Once a month or less	68 (2.7%)	174 (6.9%)	242 (9.6%)	**0.001**	
	Once a week	132 (5.3%)	429 (17.1%)	561 (22.3%)		
	Three to four times a week	225 (9.0%)	686 (27.3%)	911 (36.3%)		
	Almost daily	145 (5.8%)	652 (26.0%)	797 (31.7%)		
Fresh fruit and vegetable intake (before the start of the pandemic):	Once a month or less	16 (0.6%)	56 (2.2%)	72 (2.9%)	**0.001**	**0.000**
	Once a week	88 (3.5%)	229 99.1%)	317 (12.6%)		
	Three to four times a week	211 (8.4%)	611 (24.3%)	822 (32.7%)		
	Almost daily	255 (10.2%)	1044 (41.6%)	1299 (51.8%)		
Fresh fruit and vegetable intake (after the start of the pandemic)	Once a month or less	23 (0.9%)	69 (2.7%)	92 (3.7%)	**0.000**	
	Once a week	70 (2.8%)	184 (7.3%)	254 (10.1%)		
	Three to four times a week	193 (7.7%)	526 (21.0%)	719 (28.6%)		
	Almost daily	284 (11.3%)	1161 (46.3%)	1445 (57.6%)		
Since the start of the pandemic) have you added any of these ingredients (garlic, onion, ginger) to your diet, or increased your intake of them?	Yes	316 (12.6%)	1329 (52.9%)	1645 (65.5%)	**0.000**	
	No	254 (10.1%)	612 (24.4%)	866 (34.5%)		
(Since the start of the pandemic) have you noticed any change in your weight?	I have gained weight	212 (8.4%)	701 (27.9%)	913 (36.4%)	**0.003**	
	I have lost weight.	79 (3.1%)	394 (15.7%)	473 (18.8%)		
	My weight hasn't changed	244 (9.7%)	717 (28.6%)	961 (38.3%)		
	I don't know	35 (1.4%)	129 (5.1%)	164 (6.5%)		
(Since the start of the pandemic) has the number of meals you eat in a day changed?	increased	176 (7.0%)	668 (26.6%)	844 (33.6%)	0.226	
	decreased	91 (3.6%)	316 (12.6%)	407 (16.2%)		
	No change	303 (12.1%)	957 (38.1%)	1260 (50.2%)		
(Since the start of the pandemic) has the number of meals you eat during nighttime increased?	Yes	221 (8.8%)	784 (31.2%)	1005 (40.0%)	0.488	
	No	349 (13.9%)	1157 (46.1%)	1506 (60.0%)		
(Since the start of the pandemic) have you noticed any change in your appetite?	increased	180 (7.2%)	765 (30.5%)	945 (37.6%)	**0.001**	
	decreased	92 (3.7%)	320 (12.7%)	412 (16.4%)		
	No change	298 (11.9%)	856 (34.1%)	1154 (46.0%)		
Downloading any application or started following any social media account concerning Healthy Nutrition	Yes	122 (4.9%)	596 (23.7%)	718 (28.6%)	**0.000**	
	No	448 (17.8%)	1345 (53.6%)	1793 (71.4%)		
	No	325 (12.9%)	1708 (68.0%)	2033 (81.0%)		
Before the pandemic, were you interested in consuming nutritional supplements like vitamins and minerals including iron and zinc?	Yes	124 (4.9%)	667 (26.6%)	791 (31.5%)	**0.000**	**0.000**
	No	446 (17.8%)	1274 (50.7%)	1720 (68.5%)		
Since the beginning of the pandemic, did you introduce nutritional supplements to your diet or increase the amount that you consume?	Yes	204 (8.1%)	947 (37.7%)	1151 (45.8%)	**0.000**	
	No	366 (14.6%)	994 (39.6%)	1360 (54.2%)		
Do you think nutritional supplements play a role in boosting the immunity to fight off diseases like COVID-19?	Yes, I think	385 (15.3%)	1547 (61.6%)	1932 (76.9%)	**0.000**	
	I do not think	52 (2.1%)	106 (4.2%)	158 (6.3%)		
	I do not know	133 (5.3%)	288 (11.5%)	421 (16.8%)		
**Total**		**570 (22.7%)**	**1941 (77.3%)**	**2511 (100.0%)**		

Values are expressed as number and percentage from total respondents (**n (**%))Variables are considered significant at P-value < 0.05 and marked in bold.

**Table 4:** Food choices and eating-related behaviors before and during COVID-19 lockdown according to the social status (n = 2511).

		Social status			P value	Chi square test (P-value)
		Single	Married	Total		
Fast food intake (before the start of the pandemic):	Once a month or less	564 (22.5%)	550 (21.9%)	1114 (44.4%)	**0.000**	**0.000**
	Once a week	600 (23.9%)	314 (12.5%)	914 (36.4%)		
	Three to four times a week	288 (11.5%)	56 (2.2%)	344 (13.7%)		
	Almost daily	107 (4.3%)	32 (1.3%)	139 (5.5%)		
Fast food intake (after the start of the pandemic):	Once a month or less	857 (34.1%)	684 (27.3%)	1541 (61.4%)	**0.000**	
	Once a week	468 (18.6%)	206 (8.2%)	674 (26.9%)		
	Three to four times a week	162 (6.5%)	40 (1.6%)	202 (8.0%)		
	Almost daily	71 (2.8%)	22 (0.9%)	93 (3.7%)		
Sugar intake (before the start of the pandemic)	Once a month or less	84 (3.3%)	92 (3.7%)	176 (7.0%)	**0.000**	**0.000**
	Once a week	334 (13.3%)	295 (11.7%)	629 (25.0%)		
	Three to four times a week	532 (21.2%)	289 (11.5%)	821 (32.7%)		
	Almost daily	609 (24.3%)	276 (11.0%)	885 (35.2%)		
Sugar intake (after the start of the pandemic)	Once a month or less	122 (4.9%)	130 (5.2%)	252 (10.0%)	**0.000**	
	Once a week	382 (15.2%)	290 (11.5%)	672 (26.8%)		
	Three to four times a week	493 (19.6%)	296 (11.8%)	789 (31.4%)		
	Almost daily	562 (22.4%)	236 (9.4%)	798 (31.8%)		
Fat and oil intake (before the start of the pandemic)	Once a month or less	103 (4.1%)	99 (3.9%)	202 (8.0%)	**0.000**	**0.000**
	Once a week	291 (11.6%)	208 (803%)	499 (19.9%)		
	Three to four times a week	617 (24.6%)	330 (13.1%)	947 (37.7%)		
	Almost daily	548 (21.8%)	315 (12.5%)	863 (34.4%)		
Fat and oil intake (after the start of the pandemic)	Once a month or less	125 (5.0%)	117 (4.7%)	242 (9.6%)	**0.003**	
	Once a week	346 (13.8%)	215 (8.6%)	561 (22.3%)		
	Three to four times a week	570 (22.7%)	341 (13.6%)	911 (36.3%)		
	Almost daily	518 (20.6%)	279 (11.1%)	797 (31.7%)		
Fresh fruit and vegetable intake (before the start of the pandemic):	Once a month or less	65 (2.6%)	7 (0.3%)	72 (2.9%)	**0.000**	**0.000**
	Once a week	242 (9.6%)	75 (3.0%)	317 (12.6%)		
	Three to four times a week	532 (21.2%)	290 (11.6%)	822 (32.7%)		
	Almost daily	719 (28.6%)	580 (23.1%)	1299 (51.8%)		
Fresh fruit and vegetable intake (after the start of the pandemic):	Once a month or less	68 (2.7%)	24 (1.0%)	92 (3.7%)	**0.000**	
	Once a week	176 (7.0%)	78 (3.1%)	254 (10.1%)		
	Three to four times a week	479 (19.1%)	240 (9.6%)	719 (28.6%)		
	Almost daily	835 (33.3%)	610 (24.3%)	1445 (57.6%)		
(Since the start of the pandemic) have you added any of these ingredients (garlic, onion, ginger) to your diet, or increased your intake of them?	Yes	948 (37.8%)	697 (27.8%)	1645 (65.5%)	**0.000**	
	No	611 (24.3%)	255 (10.2%)	866 (34.5%)		
(Since the start of the pandemic) have you noticed any change in your weight?	I have gained weight	563 (22.4%)	350 (13.9%)	913 (36.4%)	**0.000**	
	I have lost weight	357 (14.2%)	116 (4.6%)	473 (18.8%)		
	My weight hasn't changed	524 (20.9%)	437 (17.4%)	961 (38.3%)		
	I don't know	115 (4.6%)	49 (2.0%)	164 (6.5%)		
(Since the start of the pandemic) has the number of meals you eat in a day changed?	Increased	596 (23.7%)	248 (9.9%)	844 (33.6%)	**0.000**	
	Decreased	299 (11.9%)	108 (4.3%)	407 (16.2%)		
	No change	664 (26.4%)	596 (23.7%)	1260 (50.2%)		
(Since the start of the pandemic) has the number of meals you eat during nighttime increased?	Yes	710 (28.3%)	295 (11.7%)	1005 (40.0%)	**0.000**	
	No	849 (33.8%)	657 (26.2%)	1506 (60.0%)		
(Since the start of the pandemic) have you noticed any change in your appetite?	My appetite has increased	636 (25.3%)	309 (12.3%)	945 (37.6%)	**0.000**	
	My appetite has decreased	297 (11.8%)	115 (4.6%)	412 (16.4%)		
	My appetite has not changed	626 (24.9%)	528 (21.0%)	1154 (46.0%)		
Downloading any application or started following any social media account concerning Healthy Nutrition	Yes	403 (16.0%)	315 (12.5%)	718 (28.6%)	**0.000**	
	No	1156 (46.0%)	637 (25.4%)	1793 (71.4%)		
Before the pandemic, were you interested in consuming nutritional supplements like vitamins and minerals including iron and zinc?	Yes	468 (18.6%)	323 (12.9%)	791 (31.5%)	**0.041**	**0.000**
	No	1091 (43.4%)	629 (25.0%)	1720 (68.5%)		
Since the beginning of the pandemic, did you introduce nutritional supplements to your diet or increase the amount that you consume?	Yes	688 (27.4%)	463 (18.4%)	1151 (45.8%)	**0.028**	
	No	871 (34.7%)	489 (19.5%)	1360 (54.2%)		
Do you think nutritional supplements play a role in boosting the immunity to fight off diseases like COVID-19?	Yes	1197 (47.7%)	735 (29.3%)	1923 (76.9%)	0.475	
	No	105 (4.2%)	53 (2.1%)	158 (6.3%)		
	I do not know	257 (10.2%)	164 (6.5%)	421 (16.8%)		
**Total**		**1559 (62.1%)**	**952 (37.9%)**	**2511 (100.0%)**		

Values are expressed as number and percentage from total respondents (**n (%)**)Variables are considered significant at P-value < 0.05 and marked in bold.

**Table 5:** Food choices and eating-related behaviors before and during COVID-19 lockdown according to the educational level of participants (n = 2511).

		Educational level:					P value	Chi square test (P-value)
		High school diploma or still in school	Diploma or bachelor's degree in a health-related major	Diploma or bachelor's degree in non-health-related major	Master's degree or PhD	Total		
Fast food intake (before the start of the pandemic):	Once a month or less	201 (8.0%)	286 (11.4%)	480 (19.1%)	147 (5.9%)	1114 (44.4%)	**0.000**	**0.000**
	Once a week	120 (4.8%)	326 (13.0%)	368 (14.7%)	100 (4.0%)	914 (36.4%)		
	Three to four times a week	42 (1.7%)	153 (6.1%)	107 (4.3%)	42 (1.7%)	344 (13.7%)		
	Almost daily	17 (0.7%)	55 (2.2%)	50 (2.0%)	17 (0.7%)	139 (5.5%)		
Fast food intake (after the start of the pandemic):	Once a month or less	239 (9.5%)	468 (18.6%)	638 (25.4%)	196 (7.8%)	1541 (61.4%)	0.117	
	Once a week	90 (3.6%)	252 (10.0%)	257 (10.2%)	75 (3.0%)	674 (26.9%)		
	Three to four times a week	32 (1.3%)	69 (2.7%)	75 (3.0%)	26 (1.0%)	202 (8.0%)		
	Almost daily	19 (0.8%)	31 (1.2%)	34 (1.4%)	9 (0.4%)	93 (3.7%)		
Sugar intake (before the start of the pandemic)	Once a month or less	27 (1.1%)	48 (1.9%)	77 (3.1%)	24 (1.0%)	176 (7.0%)	0.378	**0.000**
	Once a week	104 (4.1%)	190 (7.6%)	247 (9.8%)	88 (3.5%)	629 (25.0%)		
	Three to four times a week	116 (4.6%)	274 (10.9%)	333 (13.3%)	98 (3.9%)	821 (32.7%)		
	Almost daily	133 (5.3%)	308 (12.3%)	348 (13.9%)	96 (3.8%)	885 (35.2%)		
Sugar intake (after the start of the pandemic)	Once a month or less	47 (1.9%)	59 (2.3%)	98 (3.9%)	48 (1.9%)	252 (10.0%)	**0.001**	
	Once a week	95 (3.8%)	203 (8.1%)	288 (11.5%)	86 (3.4%)	672 (26.8%)		
	Three to four times a week	114 (4.5%)	273 (10.9%)	313 (12.5%)	89 (3.5%)	789 (31.4%)		
	Almost daily	124 (4.9%)	285 (11.4%)	306 (12.2%)	83 (3.3%)	798 (31.8%)		
Fat and oil intake (before the start of the pandemic)	Once a month or less	34 (1.4%)	51 (2.0%)	89 (3.5%)	28 (1.1%)	202 (8.0%)	**0.005**	**0.000**
	Once a week	71 (2.8%)	144 (5.7%)	218 (8.7%)	66 (2.6%)	499 (19.9%)		
	Three to four times a week	123 (4.9%)	342 (13.6%)	359 (14.3%)	123 (4.9%)	947 (37.7%)		
	Almost daily	152 (6.1%)	283 (11.3%)	339 (13.5%)	89 (3.5%)	863 (34.4%)		
Fat and oil intake (after the start of the pandemic)	Once a month or less	45 (1.8%)	60 (2.4%)	94 (3.7%)	43 (1.7%)	242 (9.6%)	**0.000**	
	Once a week	73 (2.9%)	162 (6.5%)	253 (10.1%)	73 (2.9%)	561 (22.3%)		
	Three to four times a week	123 (4.9%)	334 (13.3%)	346 (13.8%)	108 (4.3%)	911 (36.3%)		
	Almost daily	139 (5.5%)	264 (10.5%)	312 (12.4%)	82 (3.3%)	797 (31.7%)		
Fresh fruit and vegetable intake (before the start of the pandemic):	Once a month or less	15 (0.6%)	26 (1.0%)	27 (1.1%)	4 (0.2%)	72 (2.9%)	**0.000**	**0.000**
	Once a week	51 (2.0%)	138 (5.5%)	108 (4.3%)	20 (0.8%)	317 (12.6%)		
	Three to four times a week	117 (4.7%)	285 (11.4%)	334 (13.3%)	86 (3.4%)	822 (32.7%)		
	Almost daily	197 (7.8%)	371 (14.8%)	535 (21.3%)	196 (7.8%)	1299 (51.8%)		
Fresh fruit and vegetable intake (after the start of the pandemic):	Once a month or less	27 (1.1%)	24 (1.0%)	36 (1.4%)	5 (0.2%)	92 (3.7%)	**0.000**	
	Once a week	48 (1.9%)	84 (3.3%)	107 (4.3%)	15 (0.6%)	254 (10.1%)		
	Three to four times a week	102 (4.1%)	259 (10.3%)	279 (11.1%)	79 (3.1%)	719 (28.6%)		
	Almost daily	203 (8.1%)	453 (18.0%)	582 (23.2%)	207 (8.2%)	1445 (57.6%)		
(Since the start of the pandemic) have you added any of these ingredients (garlic, onion, ginger) to your diet, or increased your intake of them?	Yes	264 (10.5%)	488 (19.4%)	697 (27.8%)	196 (7.8%)	1645 (65.5%)	**0.000**	
	No	116 (4.6%)	332 (13.2%)	308 (12.3%)	110 (4.4%)	866 (34.5%)		
(Since the start of the pandemic) have you noticed any change in your weight?	I have gained weight	136 (5.4%)	294 (11.7%)	374 (14.9%)	109 (4.3%)	913 (36.4%)	**0.011**	
	I have lost weight	69 (2.7%)	175 (7.0%)	186 (7.4%)	43 (1.7%)	473 (18.8%)		
	My weight hasn't changed	140 (5.6%)	295 (11.7%)	384 (15.3%)	142 (5.7%)	961 (38.3%)		
	I don't know	35 (1.4%)	56 (2.2%)	61 (2.4%)	12 (0.5%)	164 (6.5%)		
(Since the start of the pandemic) has the number of meals you eat in a day changed?	Increased	113 (4.5%)	313 (12.5%)	336 (13.4%)	82 (3.3%)	844 (33.6%)	**0.000**	
	Decreased	63 (2.5%)	131 (5.2%)	174 (6.9%)	39 (1.6%)	407 (16.2%)		
	No change	204 (8.1%)	376 (15.0%)	495 (19.7%)	185 (7.4%)	1260 (50.2%)		
(Since the start of the pandemic) has the number of meals you eat during nighttime increased?	Yes	143 (5.7%)	363 (14.5%)	389 (15.5%)	110 (4.4%)	1005 (40.0%)	**0.019**	
	No	237 (9.4%)	457 (18.2%)	616 (24.5%)	196 (7.8%)	1506 (60.0%)		
(Since the start of the pandemic) have you noticed any change in your appetite?	My appetite has increased	136 (5.4%)	338 (13.5%)	371 (14.8%)	100 (4.0%)	945 (37.6%)	**0.027**	
	My appetite has decreased	73 (2.9%)	130 (5.2%)	167 (6.7%)	42 (1.7%)	412 (16.4%)		
	No change	171 (6.8%)	352 (14.0%)	467 (18.6%)	164 (6.5%)	1154 (46.0%)		
Downloading any application or started following any social media account concerning Healthy Nutrition	Yes	114 (4.5%)	227 (9.0%)	290 (11.5%)	87 (3.5%)	718 (28.6%)	0.864	
	No	266 (10.6%)	593 (23.6%)	715 (28.5%)	219 (8.7%)	1793 (71.4%)		
Before the pandemic, were you interested in consuming nutritional supplements like vitamins and minerals including iron and zinc?	Yes	116 (4.6%)	255 (10.2%)	310 (12.3%)	110 (4.4%)	791 (31.5%)	0.357	**0.003**
	No	264 (10.5%)	565 (22.5%)	695 (27.7%)	196 (7.8%)	1720 (68.5%)		
Since the beginning of the pandemic, did you introduce nutritional supplements to your diet or increase the amount that you consume?	Yes	175 (7.0%)	385 (15.3%)	435 (17.3%)	156 (6.2%)	1151 (45.8%)	0.097	
	No	205 (8.2%)	435 (17.3%)	570 (22.7%)	150 (6.0%)	1360 (54.2%)		
Do you think nutritional supplements play a role in boosting the immunity to fight off diseases like COVID-19?	Yes	270 (10.8%)	658 (26.2%)	765 (30.5%)	239 (9.5%)	1932 (76.9%)	**0.012**	
	No	27 (1.1%)	52 (2.1%)	59 (2.3%)	20 (0.8%)	158 (6.3%)		
	I do not know	83 (3.3%)	110 (4.4%)	181 (7.2%)	47 (1.9%)	421 (16.8%)		
**Total**		**380 (15.1%)**	**820 (32.7%)**	**1005 (40.0%)**	**306 (12.2%)**	**2511 (100.0%)**		

Values are expressed as number and percentage from total respondents (n (%))Variables are considered significant at P-value < 0.05 and marked in bold.

**Table 6:** Food choices and eating-related behaviors before and during COVID-19 lockdown according to the occupation of the participants (n = 2511).

		Occupation				P value	Chi square test (P-value)
		I work in a health-related field	I work in a non-health-related field	I don't work (student, retired, housewife, etc.)	Total		
Fast food intake (before the start of the pandemic)	Once a month or less	70 (2.8%)	276 (11.0%)	768 (30.6%)	1114 (44.4%)	**0.003**	**0.000**
	Once a week	69 (2.7%)	207 (8.2%)	638 (25.4%)	914 (36.4%)		
	Three to four times a week	40 (1.6%)	63 (2.5%)	241 (9.6%)	344 (13.7%)		
	Almost daily	17 (0.7%)	24 (1.0%)	98 (3.9%)	139 (5.5%)		
Fast food intake (after the start of the pandemic)	Once a month or less	103 (4.1%)	347 (13.8%)	1091 (43.5%)	1541 (61.4%)	0.186	
	Once a week	63 (2.5%)	155 (6.2%)	456 (18.2%)	674 (26.9%)		
	Three to four times a week	20 (0.8%)	50 (2.0%)	132 (5.3%)	202 (8.0%)		
	Almost daily	10 (0.4%)	18 (0.7%)	65 (2.6%)	93 (3.7%)		
Sugar intake (before the start of the pandemic)	Once a month or less	12 (0.5%)	44 (1.8%)	120 (4.8%)	176 (7.0%)	**0.044**	**0.000**
	Once a week	50 (2.0%)	161 (6.4%)	418 (16.6%)	629 (25.0%)		
	Three to four times a week	64 (2.5%)	199 (7.9%)	558 (22.2%)	821 (32.7%)		
	Almost daily	70 (2.8%)	166 (6.6%)	649 (25.8%)	885 (35.2%)		
Sugar intake (after the start of the pandemic)	Once a month or less	14 (0.6%)	70 (2.8%)	168 (6.7%)	252 (10.0%)	**0.000**	
	Once a week	59 (2.3%)	168 (6.7%)	445 (17.7%)	672 (26.8%)		
	Three to four times a week	72 (2.9%)	192 (7.6%)	525 (20.9%)	789 (31.4%)		
	Almost daily	51 (2.0%)	140 (5.6%)	607 (24.2%)	798 (31.8%)		
Fat and oil intake (before the start of the pandemic)	Once a month or less	16 (0.6%)	58 (2.3%)	128 (5.1%)	202 (8.0%)	**0.016**	**0.000**
	Once a week	42 (1.7%)	123 (4.9%)	334 (13.3%)	499 (19.9%)		
	Three to four times a week	82 (3.3%)	221 (8.8%)	644 (25.6%)	947 (37.7%)		
	Almost daily	56 (2.2%)	168 (6.7%)	639 (25.4%)	863 (34.4%)		
Fat and oil intake (after the start of the pandemic)	Once a month or less	15 (0.6%)	65 (2.6%)	162 (6.5%)	242 (9.6%)	**0.003**	
	Once a week	50 (2.0%)	139 (5.5%)	372 (14.8%)	561 (22.3%)		
	Three to four times a week	85 (3.4%)	211 (8.4%)	615 (24.5%)	911 (36.3%)		
	Almost daily	46 (1.8%)	155 (6.2%)	596 (23.7%)	797 (31.7%)		
Fresh fruit and vegetable intake (before the start of the pandemic)	Once a month or less	8 (0.3%)	9 (0.4%)	55 (2.2%)	72 (2.9%)	**0.034**	**0.000**
	Once a week	29 (1.2%)	54 (2.2%)	234 (9.3%)	317 (12.6%)		
	Three to four times a week	67 (2.7%)	198 (7.9%)	557 (22.2%)	822 (32.7%)		
	Almost daily	92 (3.7%)	309 (12.3%)	898 (35.8%)	1299 (51.8%)		
Fresh fruit and vegetable intake (after the start of the pandemic)	Once a month or less	7 (0.3%)	16 (0.6%)	69 (2.7%)	92 (3.7%)	0.190	
	Once a week	15 (0.6%)	47 (1.9%)	192 (7.6%)	254 (10.1%)		
	Three to four times a week	65 (2.6%)	171 (6.8%)	483 (19.2%)	719 (28.6%)		
	Almost daily	109 (4.3%)	336 (13.4%)	1000 (39.8%)	1445 (57.6%)		
(Since the start of the pandemic) have you added any of these ingredients (garlic, onion, ginger) to your diet, or increased your intake of them?	Yes	114 (4.5%)	380 (15.1%)	1151 (45.8%)	1645 (65.5%)	0.075	
	No	82 (3.3%)	190 (7.6%)	594 (23.7%)	866 (34.5%)		
(Since the start of the pandemic) have you noticed any change in your weight?	I have gained weight	65 (2.6%)	224 (8.9%)	624 (24.9%)	913 (36.4%)	**0.000**	
	I have lost weight	34 (1.4%)	83 (3.3%)	356 (14.2%)	473 (18.8%)		
	My weight hasn't changed	93 (3.7%)	232 (9.2%)	636 (25.3%)	961 (38.3%)		
	I don't know	4 (0.2%)	31 (1.2%)	129 (5.1%)	164 (6.5%)		
(Since the start of the pandemic) has the number of meals you eat in a day changed?	Increased	62 (2.5%)	175 (7.0%)	607 (24.2%)	844 (33.6%)	0.069	
	Decreased	25 (1.0%)	87 (3.5%)	295 (11.7%)	407 (16.2%)		
	No change	109 (4.3%)	308 (12.3%)	843 (33.6%)	1260 (50.2%)		
(Since the start of the pandemic) has the number of meals you eat during nighttime increased?	Yes	70 (2.8%)	196 (7.8%)	739 (29.4%)	1005 (40.0%)	**0.002**	
	No	126 (5.0%)	374 (14.9%)	1006 (40.1%)	1506 (60.0%)		
(Since the start of the pandemic) have you noticed any change in your appetite?	My appetite has increased	71 (2.8%)	191 (7.6%)	683 (27.2%)	945 (37.6%)	**0.012**	
	My appetite has decreased	30 (1.2%)	82 (3.3%)	300 (11.9%)	412 (16.4%)		
	No change	95 (3.8%)	297 (11.8%)	762 (30.3%)	1154 (46.0%)		
Downloading any application or started following any social media account concerning Healthy Nutrition	Yes	61 (2.4%)	164 (6.5%)	493 (19.6%)	718 (28.6%)	0.697	
	No	135 (5.4%)	406 (16.2%)	1252 (49.9%)	1793 (71.4%)		
Before the pandemic, were you interested in consuming nutritional supplements like vitamins and minerals including iron and zinc?	Yes	74 (2.9%)	179 (7.1%)	538 (21.4%)	791 (31.5%)	0.141	0.378
	No	122 (4.9%)	391 (15.6%)	1207 (48.1%)	1720 (68.5%)		
Since the beginning of the pandemic, did you introduce nutritional supplements to your diet or increase the amount that you consume?	Yes	106 (4.2%)	266 (10.6%)	779 (31.0%)	1151 (45.8%)	**0.038**	
	No	90 (3.6%)	304 (12.1%)	966 (38.5%)	1360 (54.2%)		
Do you think nutritional supplements play a role in boosting the immunity to fight off diseases like COVID-19?	Yes	165 (6.6%)	433 (17.2%)	1334 (53.1%)	1932 (76.9%)	**0.001**	
	No	18 (0.7%)	30 (1.2%)	110 (4.4%)	158 (6.3%)		
	I do not know	13 (0.5%)	107 (4.3%)	301 (12.0%)	421 (16.8%)		
**Total**		**196 (7.8%)**	**570 (22.7%)**	**1745 (69.5%)**	**2511 (100%)**		

Values are expressed as number and percentage from total respondents (n (%))Variables are considered significant at P-value < 0.05 and marked in bold.

**Table 7:** Association between fresh fruit and vegetable intake (after the start of the pandemic) and the other variables.

		Fresh fruit and vegetable intake (after the start of the pandemic):					P value
		Once a month or less	Once a week	Three to four times a week	Almost daily	Total	
Do you currently perform any form of physical exercise?	Yes	37 (1.5%)	77 (3.1%)	295 (11.8%)	694 (27.6%)	1103 (43.9%)	**0.000**
	No	55 (2.2%)	177 (7.1%)	424 (16.9%)	751 (29.9%)	1407 (56.1%)	
(Since the start of the pandemic) have you added any of these ingredients (garlic, onion, ginger) to your diet, or increased your intake of them?	Yes	57 (2.3%)	150 (6.0%)	437 (17.4%)	1000 (39.8%)	1644 (65.5%)	**0.000**
	No	35 (1.4%)	104 (4.1%)	282 (11.2%)	445 (17.7%)	866 (34.5%)	
(Since the start of the pandemic) have you noticed any change in your weight?	I have gained weight	38 (1.5%)	109 (4.3%)	294 (11.7%)	472 (18.8%)	913 (36.4%)	**0.000**
	I have lost weight	17 (0.7%)	47 (1.9%)	116 (4.6%)	293 (11.7%)	473 (18.8%)	
	No change	26 (1.0%)	82 (3.3%)	261 (10.4%)	591 (23.5%)	960 (38.2%)	
	I don't know	11 (0.4%)	16 (0.6%)	48 (1.9%)	89 (3.5%)	164 (6.5%)	
(Since the start of the pandemic) has the number of meals you eat in a day changed?	Increased	29 (1.2%)	100 (4.0%)	264 (10.5%)	450 (17.9%)	843 (33.6%)	**0.000**
	Decreased	25 (1.0%)	54 (2.2%)	105 (4.2%)	223 (8.9%)	407 (16.2%)	
	No change	38 (1.5%)	100 (4.0%)	350 (13.9%)	772 (30.8%)	1260 (50.2%)	
(Since the start of the pandemic) has the number of meals you eat during nighttime increased?	Yes	41 (1.6%)	123 (4.9%)	311 (12.4%)	529 (21.1%)	1004 (40.0%)	**0.000**
	No	51 (2.0%)	131 (5.2%)	408 (16.3%)	916 (36.5%)	1506 (60.0%)	
(Since the start of the pandemic) have you noticed any change in your appetite?	increased	36 (1.4%)	101 (4.0%)	297 (11.8%)	511 (20.4%)	945 (37.6%)	0.051
	decreased	21 (0.8%)	43 (1.7%)	114 (4.5%)	234 (9.3%)	412 (16.4%)	
	No change	35 (1.4%)	110 (4.4%)	308 (12.3%)	700 (27.9%)	1153 (45.9%)	
Downloading any application or started following any social media account concerning Healthy Nutrition	Yes	25 (1.0%)	60 (2.4%)	170 (6.8%)	462 (18.4%)	717 (28.6%)	**0.000**
	No	67 (2.7%)	194 (7.7%)	549 (21.9%)	983 (39.2%)	1793 (71.4%)	
Since the beginning of the pandemic, have you noticed a change with regard to your sleep hours per day?	Less	10 (0.4%)	39 (1.6%)	110 (4.4%)	180 (7.2%)	339 (13.5%)	**0.000**
	More	48 (1.9%)	128 (5.1%)	288 (11.5%)	537 (21.4%)	1001 (39.9%)	
	No change	34 (1.4%)	87 (3.5%)	321 (12.8%)	728 (29.0%)	1170 (46.6%)	
Do you currently smoke (this includes regular cigarettes, electronic cigarettes, hookah, etc.)?	Yes	20 (0.8%)	69 (2.7%)	138 (5.5%)	251 (10.0%)	478 (19.0%)	**0.003**
	No	72 (2.9%)	185 (7.4%)	581 (23.1%)	1194 947.6%)	2032 (81.0%)	
Since the beginning of the pandemic, did you introduce nutritional supplements to your diet or increase the amount that you consume?	Yes	33 (1.3%)	92 (3.7%)	315 (12.5%)	711 (28.3%)	1151 (45.9%)	**0.000**
	No	59 (2.4%)	162 (6.5%)	404 (16.1%)	734 (29.2%)	1359 (54.1%)	
Do you think nutritional supplements play a role in boosting the immunity to fight off diseases like COVID-19?	Yes	62 (2.5%)	186 (7.4%)	549 (21.9%)	1134 (45.2%)	1931 (76.9%)	0.147
	No	7 (0.3%)	17 (0.7%)	49 (2.0%)	85 (3.4%)	158 (6.3%)	
	I do not know	23 (0.9%)	51 (2.0%)	121 (4.8%)	226 (9.0%)	421 (16.8%)	
**Total**		**92 (3.7%)**	**254 (10.1%)**	**719 (28.6%)**	**1445 (57.6%)**	**2510 (100.0%)**	

Values are expressed as number and percentage from total respondents (n (%))Variables are considered significant at P-value < 0.05 and marked in bold.

**Table 8:** Association between adding or increasing intake of (garlic, onion, ginger) to the respondent's diet since the start of pandemic and the other variables.

		(Since the start of the pandemic) have you added any of these ingredients (garlic, onion, ginger) to your diet, or increased your intake of them?			P- value
		Yes	No	Total	
Do you currently perform any form of physical exercise?	Yes	750 (29.9%)	353 (14.1%)	1103 (43.9%)	**0.020**
	No	895 (35.6%)	513 (20.4%)	1407 (56.1%)	
Fast food intake (after the start of the pandemic)	Once a month or less	1058 (42.2%)	483 (19.2%)	1541 (61.4%)	**0.000**
	Once a week	412 (16.4%)	262 (10.4%)	674 (26.9%)	
	Three to four times a week	114 (4.5%)	88 (3.5%)	202 (8.0%)	
	Almost daily	60 (2.4%)	33 (1.3%)	93 (3.7%)	
Sugar intake (after the start of the pandemic)	Once a month or less	179 (7.1%)	73 (2.9%)	252 (10.0%)	**0.000**
	Once a week	474 (18.9%)	198 (7.9%)	672 (26.8%)	
	Three to four times a week	516 (20.5%)	273 (10.9%)	789 (31.4%)	
	Almost daily	476 (19.0%)	322 (12.8%)	798 (31.8%)	
Fat and oil intake (after the start of the pandemic)	Once a month or less	165 (6.6%)	77 (3.1%)	242 (9.6%)	0.594
	Once a week	369 (14.7%)	192 (7.6%)	561 (22.3%)	
	Three to four times a week	583 (23.2%)	328 (13.1%)	911 (36.3%)	
	Almost daily	528 (21.0%)	269 (10.7%)	797 (31.7%)	
Fresh fruit and vegetable intake (after the start of the pandemic)	Once a month or less	57 (2.3%)	35 (1.4%)	92 (3.7%)	**0.000**
	Once a week	150 (6.0%)	104 (4.1%)	254 (10.1%)	
	Three to four times a week	437 (17.4%)	282 (11.2%)	719 (28.6%)	
	Almost daily	1000 (39.8%)	445 (17.7%)	1445 (57.6%)	
Since the start of the pandemic) have you noticed any change in your weight?	I have gained weight	612 (24.4%)	301 (12.0%)	913 (36.4%)	0.187
	I have lost weight	321 (12.8%)	152 (6.0%)	473 (18.8%)	
	My weight hasn't changed	611 (24.3%)	350 (14.0%)	961 (38.3%)	
	I don't know	101 (4.0%)	63 (2.5%)	164 (6.5%)	
(Since the start of the pandemic) has the number of meals you eat in a day changed?	Increased	575 (22.9%)	269 (10.7%)	844 (33.6%)	0.141
	Decreased	259 (10.3%)	148 (5.9%)	407 (16.2%)	
	No change	811 (32.3%)	449 (17.9%)	1260 (50.2%)	
(Since the start of the pandemic) has the number of meals you eat during nighttime increased?	Yes	675 (26.9%)	330 (13.1%)	1005 (40.0%)	0.155
	No	970 (38.6%)	536 (21.4%)	1506 (60.0%)	
(Since the start of the pandemic) have you noticed any change in your appetite?	My appetite has increased	654 (26.0%)	291 (11.6%)	945 (37.6%)	**0.009**
	My appetite has decreased	257 (10.2%)	155 (6.2%)	412 (16.4%)	
	No change	734 (29.3%)	420 (16.7%)	1154 (46.0%)	
Downloading any application or started following any social media account concerning Healthy Nutrition	Yes	564 (22.5%)	154 (6.1%)	718 (28.6%)	**0.000**
	No	1081 (43.1%)	712 (28.4%)	1793 (71.4%)	
Do you currently smoke (this includes regular cigarettes, electronic cigarettes, hookah, etc.)?	Yes	318 (12.7%)	160 (6.4%)	478 (19.0%)	0.604
	No	1327 (52.8%)	706 (28.1%)	2033 (81.0%)	
Since the beginning of the pandemic, did you introduce nutritional supplements to your diet or increase the amount that you consume?	Yes	853 (34.0%)	298 (11.9%)	1151 (45.8%)	**0.000**
	No	792 (31.5%)	568 (22.6%)	1360 (54.2%)	
Do you think nutritional supplements play a role in boosting the immunity to fight off diseases like COVID-19?	Yes	1316 (52.4%)	616 (24.5%)	1932 (76.9%)	**0.000**
	No	92 (3.7%)	66 (2.6%)	158 (6.3%)	
	I do not know	237 (9.4%)	184 (7.3%)	421 (16.8%)	
**Total**		**1645 (65.5%)**	**866 (34.5%)**	**2511 (100.0%)**	

Values are expressed as number and percentage from total respondents (n (%))Variables are considered significant at P-value < 0.05 and marked in bold.

**Table 9:** Association between changes in weight since the start of the pandemic and the other variables.

		(Since the start of the pandemic) have you noticed any change in your weight?					P value
		I have gained weight	I have lost weight	My weight hasn't changed	I don't know	Total	
Do you currently perform any form of physical exercise?	Yes	357 (14.2%)	250 (10.0%)	425 (16.9%)	71 (2.8%)	1103 (43.9%)	**0.000**
	No	556 (22.1%)	223 (8.9%)	536 (21.3%)	93 (3.7%)	1408 (56.1%)	
Fast food intake (after the start of the pandemic):	Once a month or less	519 (20.7%)	308 (12.3%)	615 (24.5%)	99 (3.9%)	1541 (61.4%)	**0.001**
	Once a week	261 (10.4%)	129 (5.1%)	245 (9.8%)	39 (1.6%)	674 (26.9%)	
	Three to four times a week	84 (3.3%)	27 (1.1%)	74 (2.9%)	17 (0.7%)	202 (8.0%)	
	Almost daily	49 (2.0%)	9 (0.4%)	26 (1.0%)	9 (0.4%)	93 (3.7%)	
Sugar intake (after the start of the pandemic)	Once a month or less	72 (2.9%	75 (3.0%)	93 (3.7%)	12 (0.5%)	252 (10.0%)	**0.000**
	Once a week	184 (7.3%	161 (6.4%)	277 (11.0%)	50 (2.0%)	672 (26.8%)	
	Three to four times a week	302 (12.0%	129 (5.1%)	309 (12.3%)	49 (2.0%)	789 (31.4%)	
	Almost daily	355 (14.1%	108 (4.3%)	282 (11.2%)	53 (2.1%)	798 (31.8%)	
Fat and oil intake (after the start of the pandemic)	Once a month or less	64 (2.5%)	76 (3.0%)	93 (3.7%)	9 (0.4%)	242 (9.6%)	**0.000**
	Once a week	163 (6.5%)	120 (4.8%)	236 (9.4%)	42 (1.7%)	561 (22.3%)	
	Three to four times a week	327 (13.0%)	153 (6.1%)	375 (14.9%)	56 (2.2%)	911 (36.3%)	
	Almost daily	359 (14.3%)	124 (4.9%)	257 (10.2%)	57 (2.3%)	797 (31.7%)	
Fresh fruit and vegetable intake (after the start of the pandemic)	Once a month or less	38 (1.5%)	17 (0.7%)	26 (1.0%)	11 (0.4%)	92 (3.7%)	**0.000**
	Once a week	109 (4.3%)	47 (1.9%)	82 (3.3%)	16 (0.6%)	254 (10.1%)	
	Three to four times a week	294 (11.7%)	116 (4.6%)	261 (10.4%)	48 (1.9%)	719 (28.6%)	
	Almost daily	472 (18.8%)	293 (11.7%)	591 (23.5%)	89 (3.5%)	1445 (57.6%)	
(Since the start of the pandemic) has the number of meals you eat in a day changed?	Increased	549 (21.9%)	61 (2.4%)	193 (7.7%)	41 (1.6%)	844 (33.6%)	**0.000**
	Decreased	53 (2.1%)	223 (8.9%)	111 (4.4%)	20 (0.8%)	407 (16.2%)	
	No change	311 (12.4%)	189 (7.5%)	657 (26.2%)	103 (4.1%)	1260 (50.2%)	
(Since the start of the pandemic) has the number of meals you eat during nighttime increased?	Yes	583 (23.2%)	103 (4.1%)	268 (10.7%)	51 (2.0%)	1005 (40.0%)	**0.000**
	No	330 (13.1%)	370 (14.7%)	693 (27.6%)	113 (4.5%)	1506 (60.0%)	
(Since the start of the pandemic) have you noticed any change in your appetite?	My appetite has increased	618 (24.6%)	61 (2.4%)	211 (8.4%)	55 (2.2%)	945 (37.6%)	**0.000**
	My appetite has decreased	53 (2.1%)	214 (8.5%)	127 (5.1%)	18 (0.7%)	412 (16.4%)	
	No change	242 (9.6%)	198 (7.9%)	623 (24.8%)	91 (3.6%)	1154 (46.0%)	
Downloading any application or started following any social media account concerning Healthy Nutrition	Yes	249 (9.9%)	153 (6.1%)	280 (11.2%)	36 (1.4%)	718 (28.6%)	0.052
	No	664 (26.4%)	320 (12.7%)	681 (27.1%)	128 (5.1%)	1793 (71.4%)	
Do you currently smoke (this includes regular cigarettes, electronic cigarettes, hookah, etc.)?	Yes	184 (7.3%)	98 (3.9%)	170 (6.8%)	26 (1.0%)	478 (19.0%)	0.282
	No	729 (29.0%)	375 (14.9%)	791 (31.5%)	138 (5.5%)	2033 (81.0%)	
Since the beginning of the pandemic, did you introduce nutritional supplements to your diet or increase the amount that you consume?	Yes	424 (16.9%)	235 (9.4%)	434 (17.3%)	58 (2.3%)	1151 (45.8%)	**0.016**
	No	489 (19.5%)	238 (9.5%)	527 (21.0%)	106 (4.2%)	1360 (54.2%)	
**Total**		**913 (36.4%)**	**473 (18.8%)**	**961 (38.3%)**	**164 (6.5%)**	**2511 (100%)**	

Values are expressed as number and percentage from total respondents (n (%))Variables are considered significant at P-value < 0.05 and marked in bold.

**Table 10:** Association between changing in the number of meals since the start of the pandemic and the other variables.

		(Since the start of the pandemic) has the number of meals you eat in a day changed?				P value
		Increased	Decreased	No change	Total	
Do you currently perform any form of physical exercise?	Yes	335 (13.3%)	208 (8.3%)	560 (22.3%)	1103 (43.9%)	**0.001**
	No	509 (20.3%)	199 (7.9%)	700 (27.9%)	1408 (56.1%)	
Fast food intake (after the start of the pandemic)	Once a month or less	451 (18.0%)	240 (9.6%)	850 (33.9%)	1541 (61.4%)	**0.000**
	Once a week	253 (10.1%)	116 (4.6%)	305 (12.2%)	674 (26.9%)	
	Three to four times a week	93 (3.7%)	33 (1.3%)	76 (3.0%)	202 (8.0%)	
	Almost daily	46 (1.8%)	18 (0.7%)	29 (1.0%)	93 (3.7%)	
Sugar intake (after the start of the pandemic)	Once a month or less	45 (1.8%)	67 (2.7%)	140 (5.6%)	252 (10.0%)	**0.000**
	Once a week	153 (6.1%)	126 (5.0%)	393 (15.7%)	672 (26.8%)	
	Three to four times a week	280 (11.2%)	115 (4.6%)	394 (15.7%)	789 (31.4%)	
	Almost daily	366 (14.6%)	99 (3.9%)	333 (13.3%)	798 (31.8%)	
Fat and oil intake (after the start of the pandemic)	Once a month or less	43 (1.7%)	70 (2.8%)	129 (5.1%)	242 (6.9%)	**0.000**
	Once a week	163 (6.5%)	105 (4.2%)	293 (11.7%)	561 (22.3%)	
	Three to four times a week	291 (11.6%)	131 (5.2%)	489 (19.5%)	911 (36.3%)	
	Almost daily	347 (13.8%)	101 (4.0%)	349 (13.9%)	797 (31.7%)	
Fresh fruit and vegetable intake (after the start of the pandemic):	Once a month or less	29 (1.2%)	25 (1.0%)	38 (1.5%)	92 (3.7%)	**0.000**
	Once a week	100 (4.0%)	54 (2.2%)	100 (4.0%)	254 (10.1%)	
	Three to four times a week	264 (10.5%)	105 (4.2%)	350 (13.9%)	719 (28.6%)	
	Almost daily	450 (17.9%)	223 (8.9%)	772 (30.8%)	1445 (57.6%)	
(Since the start of the pandemic) have you noticed any change in your weight?	I have gained weight	549 (21.9%)	53 (2.1%)	311 (12.4%)	913 (36.4%)	**0.000**
	I have lost weight	61 (2.4%)	223 (8.9%)	189 (7.5%)	473 (18.8%)	
	My weight hasn't changed	193 (7.7%)	111 (4.4%)	657 (26.2%)	961 (38.3%)	
	I don't know	41 (1.6%)	20 (0.8%)	103 (4.2%)	164 (6.5%)	
(Since the start of the pandemic) has the number of meals you eat during nighttime increased?	Yes	666 (26.5%)	93 (3.7%)	246 (9.8%)	1005 (40.0%)	**0.000**
	No	178 (7.1%)	314 (12.5%)	1014 (40.4%)	1506 (60.0%)	
(Since the start of the pandemic) have you noticed any change in your appetite?	My appetite has increased	644 (25.6%)	53 (2.1%)	248 (9.9%)	945 (37.6%)	**0.000**
	My appetite has decreased	35 (1.4%)	230 (9.2%)	147 (5.9%)	412 (16.4%)	
	No change	165 (6.6%)	124 (4.9%)	865 (34.4%)	1154 (46.0%)	
Downloading any application or started following any social media account concerning Healthy Nutrition	Yes	248 (9.9%)	143 (5.7%)	327 (13.0%)	718 (28.6%)	**0.001**
	No	596 (23.7%)	264 (10.5%)	933 (37.2%)	1793 (71.4%)	
Do you currently smoke (this includes regular cigarettes, electronic cigarettes, hookah, etc.)?	Yes	184 (7.3%)	92 (3.7%)	202 (8.0%)	478 (19.0%)	**0.001**
	No	660 (26.3%)	315 (12.5%)	1058 (42.1%)	2033 (81.0%)	
Since the beginning of the pandemic, did you introduce nutritional supplements to your diet or increase the amount that you consume?	Yes	409 (16.3%)	185 (7.4%)	557 (22.2%)	1151 (45.8%)	0.156
	No	435 (17.3%)	222 (8.8%)	703 (28.0%)	1360 (54.2%)	
**Total**		**844 (33.6%)**	**407 (16.2%)**	**1260 (50.2%)**	**2511 (100%)**	

Values are expressed as number and percentage from total respondents (n (%))Variables are considered significant at P-value < 0.05 and marked in bold.

#### 3.1.4 Health-related applications and social media behaviors

When we inquired about downloading any applications or starting to follow any social media accounts concerning healthy lifestyle and nutrition during the pandemic period, 28.6% (718) of respondents stated that they have downloaded apps and/or followed such accounts. There was a strong significant association between downloading these apps and age above 35 years, female gender, married social status, eating fresh fruits and vegetables almost daily, and those who decreased the number of meals during the day (P-value = 0.000). However, there is no statistical association with occupation, educational level, and changing weight during the pandemic (P-value was 0.697, 0.864, and 0.052, respectively) [Tables 2, 3, 4, 5, 6, 7, 9, and 10].

#### 3.1.5 Nutritional supplements behaviors

One of the crucial topics brought into consideration during the pandemic is the consumption of nutritional supplements, which has increased dynamically in the wake of COVID-19. Respondents were asked about their interest in taking dietary supplements such as zinc and iron before the pandemic compared to after its onset. 31.5% (791) of the total sample said that they had already been taking supplements before the start of the pandemic), this figure rose to 1511 respondents (45.8%) taking supplements after it started, which means that this pandemic increased was taking supplements by 1.9-fold (P-value < 0.05).

Respondents that do take dietary supplements tend to have high fresh fruit & vegetable intake after the pandemic began (P-value of 0.000); as most of them fall into the "Almost daily" (28.3%) or the "Three to four times a week" (12.5%) categories. Furthermore, respondents who take supplements also tend to add onion, garlic, and ginger to their diet at the start of the pandemic (P-value 0.000). These comprise 853 (34.0%) of the respondents. Also, we found a significant association between taking dietary supplements during the pandemic with female gender (P-value 0.000), married social status (P-value = 0.028), working in a health-related field (P-value 0.038), and changes in weight during the pandemic (P-value 0.016) [Tables 3, 4, 6, 7, 8, and 9].

On the other hand, there was no relationship between taking dietary supplements with age, education, and the number of meals during pandemic days (P-values were 0.130, 0.097, and 0.156, respectively) [Tables 2, 5, and 10].

In addition to asking respondents whether they consumed dietary supplements or not, we asked whether respondents thought that nutritional supplements play a role in boosting the immunity to fight off diseases like COVID-19. Of the 2511 respondents, 1932 respondents (76.9%) believed that taking dietary supplements does help strengthen immunity. On the other hand, 158 people (6.3%) thought that the supplements did not affect them. The remainder of the respondents, who account for 16.8% (421 people) of the sample, replied with "I don't know." In conclusion, we found that those who consume nutritional supplements believe in protecting them from the disease.

Respondents who thought that nutritional supplements play a significant role in boosting immunity were strongly associated with the female gender, working in a health-related field, and respondents who added garlic, onion, and ginger ingredients to their diet (P-value for all <0.05). On the contrary, neither age, social status, nor consuming fresh fruit and vegetable intake had a statistical association (P-value > 0.05).

Moreover, the change in consumption of nutritional supplements before and after the pandemic was significantly associated with demographical factors like age, gender, social status, and educational level (P-value for all <0.05). However, there was no significant association with occupational status (P-value = 0.378) [Tables 2, 3, 4, 6, 7, and 8].

#### 3.1.6 Factors that affect eating behaviors during the pandemic

When we asked about the factors that affected eating habits and diet during the pandemic, 1439 (57.3%) respondents stated that the extra free time due to lockdown and studying or working from home had significant effects. In comparison, 926 (36.9%) realize the importance of a healthy and balanced diet in strengthening the immune system against diseases, including COVID-19, representing the health aspect. Likewise, 789 (31.4%) reported that the economic elements, such as being off work or losing a job, limited food products, and the closing of restaurants and cafes, tend to a healthier lifestyle. Only 657 (26.2%) found that the influence of family members, friends, physicians, or social media affected their eating habits during the pandemic.

## 4 Discussion

Since the beginning of the COVID-19 pandemic, Jordan has been one of many countries around the world that have introduced a new set of rules to curb the spread of COVID-19. The Jordanian government announced a national lockdown on March 17, which later turned into a strict curfew. For six weeks, the lockdown included a ban on the use of all forms of transportation and movement except for health service providers and essential sector workers. Neighborhoods with confirmed cases have been isolated, and travel between provinces is prohibited. Such strict measures mean that most Jordanians have been staying in their homes for some time, enough to bring about changes in their lifestyle and behaviors. This study better understood the eating habits and dietary patterns of Jordanians before and after the pandemic.

Our results showed a significant reduction in the rate of eating fast food and increased sugar uptake after the pandemic (P-value < 0.05). This was in line with the findings of Chopra and colleagues, who conducted their research in India and concluded that intake of unhealthy food items such as fast food, fried foods, fast food and sugar-sweetened beverages decreased significantly during COVID-19. [21]. On the other hand, this came in disagreement with Ammar, A, and colleagues who linked the epidemic to increased consumption of unhealthy foods, uncontrolled eating, snacking between meals and an increase in the total number of main meals [22]. Also, Al-Hourani H et al. Al, who conducted their research in Jordan among children and adolescents, and Kolokotroni O et al, who conducted their research in Cyprus, reported increased consumption of healthy and unhealthy food [12, 23]. Moreover, Androutsos O et al reported increased consumption of fruits, vegetables, carbohydrates and sweets while finding a significant decrease in consumption of fast food among Greek children [24].

Respondents were also asked how many meals they ate per day and whether the number of meals they ate during the night had increased during the pandemic. 844 (33.6%) of the participants noticed an increase in the number of meals they ate per day, and 549 (65.0%) gained weight. 1005 (40.0%) answered that they noticed an increase in the number of meals eaten during the night and that 583 (58.0%) of them had gained weight. This agrees with the Italian study by Mattioli, A.V, and colleagues, which states that obesity is likely to be a consequence of quarantine stress attributable to a change in lifestyle and eating habits [25]. Kenđel Jovanović G et al found significant weight gain in Croatian children as the prevalence of obesity in school children increased from 21% before closure to 24% after closure [26]. In our study, 59 out of 151 children (39.1%) gained weight during closure. This is similar to the results of Androutsos O et al who found that 35% of Greek children gained weight [24], Al Labadi et al who found that 41.7% of Palestinian children gained weight [27], and Hourani H et al. Al, who found a significant increase in the weight of Jordanian children and a wide shift from normal weight to obesity or overweight during lockdown [12]. This weight gain was associated with increased home-cooked foods, the number of meals, breakfast frequency, consumption of sweets and dairy products, time spent in front of a screen, hours of sleep, and decreased physical activity [12, 24, 27].

As for the consumption of fats and oils, the results indicated a decrease in their total consumption during the pandemic. It should be noted that the questionnaire clearly stated that it asked about unhealthy fats and oils such as vegetable oils. The group with the highest percentage of participants at 947 before the pandemic and 911 after the pandemic, which represented 37.7% and 36.3% of the survey population, respectively, had fats and oils in their diet three to four times a week (P-value < 0.05). However, in general, the figures show that there was a slight decrease in the frequency of intake of fats and oils; The number of participants who consumed fats and oils almost daily decreased from 863 before the pandemic to 797 after the onset of the pandemic; Consequently, this resulted in higher numbers falling into the less frequent consumption categories of 'once a week' and 'once a month or less.

By comparison, the Polish study entitled Feeding Behaviors of Polish adults before and during the COVID-19 lockdown found an overall increase in the consumption of oils and margarine by the Polish population during the lockdown in their country. The group with the highest percentage of participants in their study was "a few times a week" in terms of the frequency of oil and margarine intake.

When examining the fresh fruit and vegetable intake results, we found that the "Almost daily" category had the highest number of participants before and after the pandemic onset. Furthermore, there was an overall increase in the "almost daily" category by 5.8% after the pandemic (P-value < 0.05). This is going in line with the findings in a study of dietary behaviors of the Spanish adult population resulted in outlined healthier nutritional behaviors (e.g., decreased intake of fried foods, snacks, fast foods, red meat, pastries, or sweet beverages, but increased olive oil, vegetables, fruits or legumes) during the confinement during the COVID-19 outbreak when compared to previous habits [29]. Also, several studies reported increased fruit and vegetable intake during the lockdown [10, 12, 21, 24, 26]. On the contrary, Matsungo TM et al, who conducted their research in Zimbabwe, found that 64.9% and 48.5% of their participants decreased in fruit and vegetable intake respectively while they found 33.7% of participants increased dark green vegetable consumption [30]. Likewise, Mitchell SE et al found a reduction in fruit and vegetables intake among the USA population during the first week of lockdown, especially among females and young participants [31].

The least chosen category for consuming fresh fruits and vegetables was "once a month or less." Still, this category showed an increase in the percentage of participants from 2.9% before the Covid19 outbreak to 3.7% after. This indicates that people headed either towards the least frequent category or to the most frequent category (P-value < 0.05). This can be attributed to the fact that when participants were asked about their motives for their dietary choices, 926 (36.9%) responded, saying that they had realized the importance of a healthy and balanced diet in strengthening the immune system against diseases including COVID-19. Pietrobelli et al. studied a sample of 41 children and adolescents with obesity from Verona, Italy, and reported no changes in vegetable intake and increased fruit intake (p = 0.055) during the lockdown [32]. The current study showed a significant association between the consumption of fresh fruits, vegetables, fat oils, sugars, and fast food with the varying demographic characteristics measured in the survey (P-value < 0.05).

In our study, females consumed significantly more sugar, fat, oil, fruits and vegetables daily than males (p > 0.05). Also, females significantly increased their intake of ingredients such as garlic, onion and ginger, took nutritional supplements during the pandemic, downloaded any apps, or started following any social media accounts related to a healthy lifestyle and nutrition (P = 0.000). However, males were significantly consuming fast food almost daily and gained more weight compared to females since the beginning of the epidemic (P-value was 0.000 and 0.000, respectively). This means that females had significantly more healthy eating and feeding habits during the epidemic than males. Consistent with our observations, Kenđel Jovanović G et al found that females consumed significantly more olive oils and sweets than males (P-value was 0.047 and 0.002, respectively) while no association was found between gender and fruit and vegetable intake [26]. Rodríguez-Pérez C reported that males had a significant difference in the consumption of fruits, vegetables and olive oil [29]. Moreover, Poelman MP et al found that participants who were obese, overweight, had a high level of education, and were younger adults were more likely to consume an unhealthy diet [33]. Hourani and others. al reported that females consumed more food, fried foods, and less sugary drinks than males. While they did not find a significant difference between gender with sweets consumption, changes in examination time, and weight change [27].

In addition, the results revealed that the two choices with the highest response rates were "My weight hasn't changed" (38.3%), followed by "I have gained weight" (36.4%). 18.8% of participants said they had lost weight since the pandemic, whereas the rest weren't aware of any changes to their weight. Similar findings were observed in the USA-based questionnaire by Zeigler Zachary and colleagues. They found that nearly 59% of the sample reported they had remained relatively weight stable, while 22% say they have gained five to ten pounds thus far. 15% say they have lost five to ten pounds, and 4% say they have lost more than ten pounds [34]. To further emphasize, in a survey conducted by Sidor and Rzymski in a group of young people (the average age: 27.7 ± 9.0), the majority of them were women (95.1%). They reported that almost half of them hadn't observed any changes in their body weight since the pandemic onset [35]. In our study, gaining weight during the pandemic was significantly associated with age less than 18 years, male gender, married social status, having 7 or more household members, having a diploma or bachelor's degree in non-health-related major, working in a non-health-related field, not performing physical exercise, increased number of daily meals, and nighttime meals. Our results were not in complete agreement with those of Reyes-Olavarría D et al who found that their Chilean participants, who gained weight during the pandemic, were significantly associated with female gender, married, or separated marital status, moderate economic status, consumption of fried food or fast food more than 2 times per week [36]. Also, Sidor and Rzymski found that age more than 36 years, overweight, obese, consuming more food especially fast food, less fruit and vegetable intake, and more daytime meals were associated with gain weight. On the other hand, they reported no significant association with gender, educational level, and occupation (P-value > 0.05) [35].

Compared to similar Jordanian studies, Al-Domi H et al. found that Jordanian participants of the female gender, with a bachelor's degree or higher education level, who worked in any field and were chronically ill were significantly obese or overweight during the pandemic [37]. ]. Also, about three-quarters of the significantly overweight Jordanian participants had chronic diseases such as hypertension, diabetes, and heart disease [37]. Accordingly, based on a study of Jordanian COVID-19 patients, those with chronic diseases have a high risk of developing severe symptoms of COVID-19 (P-value was 0.0001) and length of hospitalization [38].

We also found it appropriate to ask participants about any changes in their appetite that coincide with the generality of religion. Nearly half of the participants (46%) said they saw no change in their appetite. On the other hand, 37.6% reported an increase in their appetite, while the remaining percentage experienced a decrease in appetite. Another study that was interested in examining people's appetite during the COVID-19 lockdown was an Italian study by Laura Di Renzo et al., and they found that the feeling of hunger and satiety changed in more than half of the population: 17.8% of the respondents had a decrease in appetite, while 34.4% of the respondents experienced an increased appetite [39].

In the present study, when cross-tabulating between changes in weight of respondents and changes in their appetites' it was found that of the 1154 individuals that experienced no alterations to their appetite, 623 didn't notice any difference in their weight either. The same pattern applied to the 945 persons who reported a growing appetite during the pandemic, 618 of them had detected a more significant number on the scale. This comes to show that in most cases, weight and appetite go hand in hand. On the contrary, Al-Domi H et al found an increased appetite among 44.3% of their Jordanian sample, 32.0% with no change, and the rest (23.6%) said “maybe” increase in their appetite. They found a significant association between increasing appetite and being obese or overweight compared to normal-weight participants (50.2%, 44.4%, and 40.4%, respectively) [37]. In our results, increases in appetite were significantly associated with respondents who were less than 18 years, female gender, single social status, had a diploma or bachelor's degree in a health-related major, had no occupation, free chronic disease, not performing physical exercise, gained weight, increased number of daily meals, and nighttime meals. Our findings concurred with those of Di Renzo L et al who found that young, female gender, suspension, or online working were significantly associated with increased appetite [39].

Studies show that the use of ginger, garlic and onion extract can have beneficial effects on individuals with lung problems such as acute respiratory distress syndrome, lung injury, pulmonary fibrosis, and pneumonia, as well as those with inflammatory conditions such as sepsis, all of which are symptoms seen in patients with COVID-19 [40].

In light of the alternative medicine recommendations circulating on social media here in Jordan, such as adding ingredients such as garlic, onions, and ginger to one's daily diet. We found that there was a significant relationship (p-value = 0.000) between those who increased the intake of the above components and those who ate a large number of fresh fruits and vegetables since the beginning of the epidemic. In our study, 65.5% of participants added at least one type of garlic, onion, and ginger to help them cope with the epidemic. On the other hand, Zhang J et al found that only 9.8% of Chinese participants added ginger to deal with the epidemic [41].

Since the start of the pandemic, there has been a constant public debate about the role of nutritional supplements in boosting the immune response against emerging diseases. Mayasari, N. et al. found a significantly increased Google search using nutritional supplements keywords especially vitamins, herbs, and zinc during the COVID-19 pandemic [42]. In systemic review found that vitamins, zinc, selenium, and probiotics have a favorable effect on strengthening immunity against the COVID-19 virus [43].

Respondents in our study were asked about their interest in taking dietary supplements such as zinc and iron before the pandemic compared to after its onset. 31.5% (791) of the total sample said that they had already been taking supplements before the pandemic; this figure rose to 1511 respondents (45.8%) taking supplements after it started. This 1.9-fold increase indicates the pandemic reinforces this dietary choice. In the Polish survey by Błaszczyk-Bebenek et al., no significant differences were noted in using dietary supplements between two-time points in the study group (p = 0.3057). Before lockdown, just over a third of the participants had used supplements, and 33.7% used them during the lockdown. The most frequently used supplement among participants was vitamin D (15.7% before and 13.5% during confinement; p = 0.2810). Magnesium was used the least frequently (2.2% before and 1.9% during confinement; p = 0.4545) [28]. Likewise, Zhang J et al reported that Chinese participants intake vitamin C (25.2%), probiotics (12.9%), ginger (9.8%), and alcohol (5.3%) to help them in coping against COVID-19 [41].

## 5 Conclusions

In conclusion, our study showed a significant reduction in sugar intake, increased appetite, weight and intake of multiple meals, fruits and vegetables per day during the COVID-19 pandemic. Moreover, the study showed some health behaviors in specific population groups related to educational level, age, and the influence of family members and friends. Some other factors related to the COVID-19 epidemic, such as smoking and drinking habits, can be investigated.

## 6 Limitations

The main limitation of this study is that it is based on a self-reported electronic questionnaire, which can lead to the possibility of reporting bias. Although this is a known problem for any online survey, we try to overcome it with a large representative sample and using the appropriate statistical tools mentioned in the methodology part. The lower number of males in our sample compared to females is another limitation.

## Declarations

### Author contribution statement

Almu'atasim Khamees: Conceived and designed the experiments; Performed the experiments; Analyzed and interpreted the data; Contributed reagents, materials, analysis tools or data; Wrote the paper.

Sajeda Awadi: Conceived and designed the experiments; Performed the experiments; Wrote the paper.

Shireen Rawashdeh, Muna Talafha, Jamal Bani-Issa: Performed the experiments; Wrote the paper.

Mohammad Ali S. Alkadiri, Mazhar Salim Al Zoubi, Emad Hussein, Fadi Abdel Fattah, Ibrahim H. Bashayreh, Mohannad Al-Saghir: Performed the experiments; Analyzed and interpreted the data; Contributed reagents, materials, analysis tools or data; Wrote the paper.

### Funding statement

This research did not receive any specific grant from funding agencies in the public, commercial, or not-for-profit sectors.

### Data availability statement

Data included in article/supplementary material/referenced in article.

### Declaration of interests statement

The authors declare no conflict of interest.

### Additional information

No additional information is available for this paper.
